# Thyroid hormone levels among adults in India: A cross-sectional study

**DOI:** 10.6026/973206300213256

**Published:** 2025-09-30

**Authors:** Hemraj, Varun Gupta, Mudassar Ahmed Shariff, Heena Dixit, Anil Tiwari, Anil Managutti, Deepak Sharma

**Affiliations:** 1 Department of General Medicine, FH Medical College and Hospital, Agra, Uttar Pradesh, India; 2 Department of Otorhinolaryngology, Chamarajanagar Institute of Medical Sciences, Chamarajanagar, Karnataka, India; 3 Department of Medical Health Administration, Index Institute, Malwanchal University, Indore, Madhya Pradesh, India; 4 Department of Oral and Maxillofacial Surgery, Narsinhbhai Patel Dental College and Hospital, Sankalchand Patel University, Visnagar, Gujarat, India

**Keywords:** Thyrotropin, thyroxine, triiodothyronine, reference values, sex characteristics

## Abstract

Age and sex influence thyroid physiology, but India-specific, age- and sex-stratified distributions for thyroid-stimulating hormone (TSH), free thyroxine (FT4) and free triiodothyronine (FT3) remain variably defined. Hence, we conducted a multicentre, community-based cross-sectional study across five Indian regions, enrolling euthyroid adults without known thyroid disease. Morning fasting samples were assayed on standardized chemiluminescent platforms; nonparametric percentiles were used to derive reference limits and regression models quantified associations. Among 5,812 adults (52.4% women; median age 41 years), median TSH was higher in women than men and increased with age, while FT4/FT3 declined modestly with age. The 97.5th percentile for TSH rose from ~4.3-4.6 mIU/L in 18-29 year-olds to ~6.1-6.4 mIU/L in those ≥60 years, with consistently higher upper limits in women. Thus, we show age- and sex-specific interpretation of thyroid tests in Indian adults.

## Background:

Inter-individual variation in thyroid function is influenced by age, sex, iodine nutrition, assay platform and population factors, limiting the utility of a single universal reference range [1]. In older adults, TSH tends to rise while FT4 remains relatively stable, raising concerns that fixed cut-offs may lead to overdiagnosis of subclinical hypothyroidism [2]. Moreover, risk-based analyses suggest that ranges associated with optimal cardiovascular and mortality outcomes may not correspond with manufacturer-provided intervals [3]. Assay differences further complicate interpretation, as switching immunoassay platforms can shift reference limits, highlighting the need for local verification [4]. Comparative work also shows that manufacturer inserts may diverge from intervals derived by modern direct or indirect methods [5]. Indian evidence remains scarce, though recent studies demonstrate feasibility of locally establishing intervals [6]. Given improved yet regionally variable iodine status [7, 8, 9- 10]. Therefore, it is of interest to define age- and sex-specific distributions of TSH, FT4 and FT3 among Indian adults.

## Materials and Methods:

## Study design and setting:

We conducted a multicentre, community-based cross-sectional study (January-December, one calendar year) across five Indian zones (North, South, East, West and Central). Sites included one urban and one peri-urban/rural field centre per zone.

## Participants:

Adults aged ≥18 years were recruited by stratified community outreach.

## Exclusion criteria:

Self-reported or documented thyroid disease, prior thyroid surgery or radioiodine, current thyroid hormone/antithyroid drugs, amiodarone/glucocorticoid/lithium use, pregnancy/lactation, acute illness or hospitalization in the prior month, eGFR <60 mL/min/1.73 m^2^, known pituitary disease, or positive anti-thyroid peroxidase antibodies (>35 IU/mL) on screening.

## Sample size:

Assuming nonparametric estimation of 2.5th/97.5th percentiles with ±0.1 mIU/L precision for TSH within age-sex strata and allowing for ~10% exclusions, a target of ≥5,500 analyzable participants was set.

## Procedures and assays:

Fasting (08:00-10:30 h) venous blood was collected once per participant. Serum TSH (mIU/L), FT4 (ng/dL) and FT3 (pg/mL) were measured on traceable chemiluminescent platforms at two central laboratories under ISO 15189 quality systems with internal QC (two levels, daily) and external proficiency participation. Cross-platform commutability panels were used before study initiation. Anthropometry and lifestyle data were recorded using standardized forms.

## Statistical analysis:

Analyses used R 4.3. Continuous variables are summarized as medians (IQR). Reference limits used the nonparametric 2.5th-97.5th percentiles after outlier filtering (Tukey fences) within age-sex strata (18-29, 30-44, 45-59, ≥60 years). Group comparisons used Wilcoxon rank-sum or Kruskal-Wallis tests with Dunn's adjustment. Associations of age (continuous) with hormones used quantile regression (median) and linear regression (log-TSH), adjusted for sex, BMI, region, smoking and alcohol. Trend across age categories used Cuzick's test. A two-sided p<0.05 was significant.

## Results:

A total of 6,214 adults were screened, of whom 402 (6.5%) were excluded, leaving 5,812 participants (52.4% women) for analysis. Median age was 41 years and median BMI 24.7 kg/m^2^. Overall median values were: TSH 2.36 mIU/L, FT4 1.18 ng/dL and FT3 3.14 pg/mL (Table 1 - see PDF). Women showed higher TSH than men (2.52 vs 2.18 mIU/L; p<0.001) and slightly lower FT4. TSH increased progressively with age, while FT4 and FT3 declined (p-trend<0.001). In adjusted models, each 10-year age increase corresponded to a 5.4% higher TSH and 0.02 ng/dL lower FT4. Age- and sex-specific reference intervals confirmed these trends: in men, upper TSH limits rose from 4.30 mIU/L at 18-29 years to 6.08 at ≥60 years; in women, from 4.58 to 6.37 mIU/L (Table 2 - see PDF). Median FT4 fell from ~1.22 to 1.13 ng/dL and FT3 from ~3.32 to 2.88 pg/mL. Applying fixed cut-offs (4.0-4.5 mIU/L) would misclassify many older adults, especially women. Regional patterns were consistent, with slightly higher TSH in northern sites.

## Discussion:

This study provides one of the largest datasets describing age- and sex-specific thyroid hormone distributions in Indian adults. The principal findings were a progressive rise in TSH with age and consistently higher TSH levels in women compared with men, while FT4 and FT3 declined modestly across age categories. These patterns have important implications for clinical interpretation of thyroid function tests in India. Age-related elevation in TSH is increasingly recognized as a physiological adaptation rather than an indicator of overt thyroid failure. Large cross-sectional studies in older adults have shown that TSH reference intervals extend well above 6 mIU/L in individuals over 70 years [11]. Similar findings were observed in Japanese and Chinese cohorts, where gradual age-related increases in TSH were evident even after excluding individuals with autoimmune thyroid disease [12]. Our results confirm these observations in an Indian context, supporting the concept of age-adjusted diagnostic cut-offs to avoid overdiagnosis of subclinical hypothyroidism in the elderly [13]. The decline in FT4 and FT3 with age complements the observed TSH rise. Prior work suggests that reduced thyroidal reserve, altered hormone metabolism and changes in feedback sensitivity contribute to these shifts [12]. Clinical consequences may be limited, since outcome studies indicate that mildly elevated TSH in older adults is not consistently associated with adverse metabolic or cardiovascular risk [15]. These findings caution against uniform levothyroxine initiation in asymptomatic elderly patients with marginal TSH elevations. Sex differences in thyroid function were also consistent. Women demonstrated higher TSH and lower FT4 compared with men across all age groups, a phenomenon also reported in European and Asian studies [9, 14]. These differences likely reflect a combination of estrogen effects, sex-related variations in binding proteins and higher prevalence of thyroid autoimmunity in women [15, 16-17]. Notably, even after excluding antibody-positive individuals, the difference persisted, suggesting intrinsic sex dimorphism. Pregnancy studies further support the biological basis of sex effects, with maternal thyroid parameters varying subtly by fetal sex [18]. Our use of nonparametric percentile estimation provides robust, population-specific reference values. International studies highlight the limitations of relying on manufacturer intervals, which may not reflect local populations or assay characteristics [13, 19]. Indirect data-mining approaches similarly demonstrate clinically meaningful differences in derived reference limits [20]. Together, these findings underscore the need for laboratories in India to adopt locally verified intervals.

## Conclusion:

TSH rises with age and is modestly higher in women, whereas FT4/FT3 decline slightly. Age-and sex-stratified reference limits (particularly a higher upper TSH limit in older adults) can sharpen interpretation and reduce overdiagnosis. Local verification on contemporary platforms is recommended.
